# 377. Impact of VE303, a Defined Bacterial Consortium, on Antimicrobial Resistant Gene Prevalence in Patients with *Clostridioides difficile* Infection (CDI)

**DOI:** 10.1093/ofid/ofae631.008

**Published:** 2025-01-29

**Authors:** Rajita Menon, Emily Crossette, Shakti Bhattarai, Vanni Bucci, Marlena Keisler, Gregory Medlock, Bernat Olle, Jeffrey L Silber, Jason Norman

**Affiliations:** Vedanta BIosciences, Cambridge, MA; Vedanta Biosciences, Somerville, Massachusetts; UMASS Chan Medical School, 368 Plantation St, Massachusetts; UMass Chan Medical School, Worcester, Massachusetts; Vedanta Biosciences, Somerville, Massachusetts; Vedanta Biosciences, Somerville, Massachusetts; Vedanta Biosciences, Somerville, Massachusetts; Vedanta Biosciences, Somerville, Massachusetts; Vedanta Biosciences, Somerville, Massachusetts

## Abstract

**Background:**

Repeated antibiotic treatment for recurrent CDI (rCDI) may promote the colonization of antimicrobial resistant (AR) microbes, which can worsen CDI outcomes and increase the risk of drug-resistant infections. Fecal microbiota products promote a gut environment resistant to CDI, but these treatments have inherently variable quality attributes, are difficult to scale, and can transfer emerging pathogens. VE303 is a defined consortium of 8 purified, clonal bacterial strains, overcoming these limitations. In the CONSORTIUM Study (NCT03788434), VE303 high-dose (HD) was well tolerated, reduced the odds of rCDI by > 80% compared with placebo, and led to both early restoration of the native microbiota and robust colonization of VE303 strains (Fig. 1A,C). VE303 strain detection was associated with clinical efficacy (Fig. 1B). Here, we assessed the resistome of subjects with CDI to examine changes in AR gene (ARG) profiles due to VE303 treatment.

**Figure 1.**
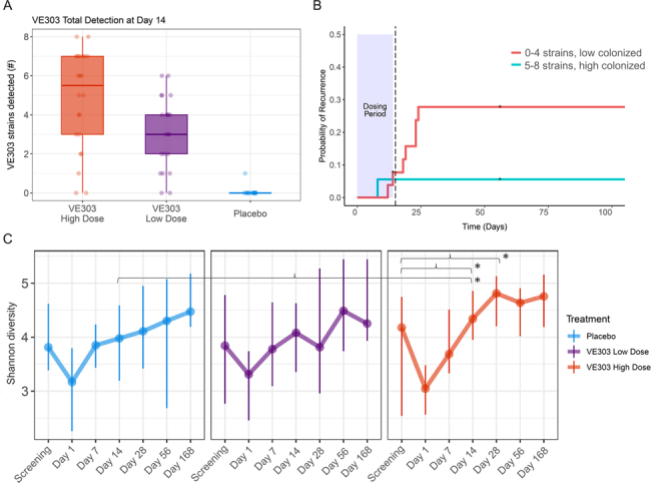
(A) Total VE303 strain detection at the end of dosing was significantly increased in VE303-dosed groups compared with the placebo group (p < 0.001, Wilcoxon test). (B) In VE303 recipients, high colonization (5 to 8 strains colonized) at the end of dosing was associated with a lower probability of recurrence (log-rank test, p=0.08, hazard ratio for low colonization/high colonization = 5.32). (C) Species alpha diversity represented by the Shannon Index over time across treatment groups. The thick lines show the median diversity across subjects at each timepoint, and the error bars show the median absolute deviation. Star and bracket annotations indicate significant differences between treatment groups at the indicated timepoints (* p < 0.05; LME model).

**Methods:**

After completing standard-of-care (SoC) antibiotic treatment for a lab-confirmed CDI episode, 79 subjects were randomized to placebo, VE303 low-dose (LD), or VE303 HD and dosed orally once daily for 14 days (Fig. 2). Subjects were followed for 24 weeks to monitor safety and rCDI episodes; samples were collected during dosing and at weeks 4 and 8 for metagenomics analysis. ARG prevalence was evaluated by aligning host-depleted reads against markers from the Comprehensive Antibiotic Resistance Database v3.2.9 using the ShortBRED pipeline v0.9.4; bacterial correlations with ARGs and changes over time were assessed by linear mixed effects (LME) models.

**Figure 2.**
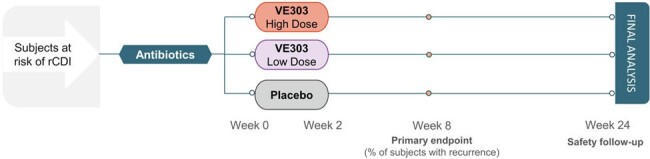
Design of the CONSORTIUM Study (NCT03788434).

**Results:**

ARG prevalence was decreased in the HD group after dosing compared to Day 1 i.e., post-SoC antibiotic treatment, for both total ARGs (Fig. 3A, p < 0.005, LME) and for the subset of ARGs positively correlated with Proteobacteria (Fig. 3B, p < 0.0005, LME). No significant changes in total or Proteobacteria-related ARGs were observed in LD or placebo subjects after dosing. Total ARG prevalence was also decreased by Day 28 in subjects with high VE303 strain colonization, regardless of the dose received (Fig. 4, p < 0.05, LME).

**Figure 3.**
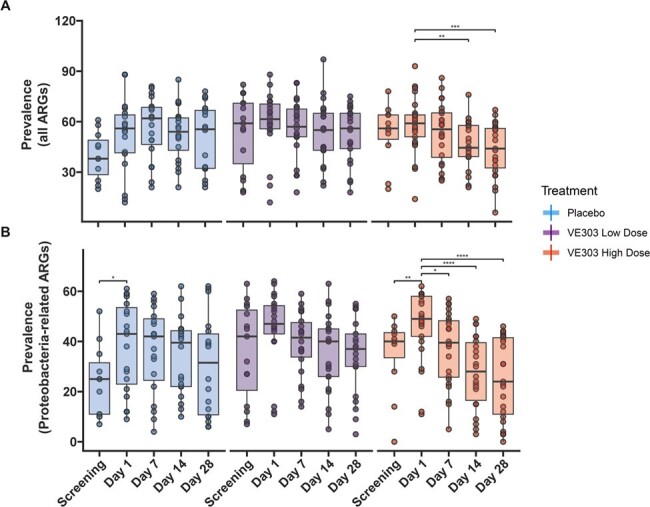
ARG prevalence in Placebo, VE303 LD and VE303 HD subjects over time for (A) all detected ARGs with non-zero RPKM (reads per kilobase per million reads) and (B) the subset of detected ARGs found to be significantly positively correlated with endogenous Proteobacteria (p < 0.05, LME). The box-and-whisker plots depict the median, interquartile range (IQR, at the top and bottom of the boxes), and reasonable extreme values at 1.5 X IQR in the dataset (where the vertical lines end). Star and bracket annotations indicate all pairwise comparisons with ARG prevalence at Day 1 that were significantly different; * p < 0.05, ** p < 0.005, *** p < 0.0005, **** p < 0.00005, LME model.

**Conclusion:**

In addition to its effects in preventing rCDI, microbiota restoration by VE303 may diminish the reservoir of drug-resistant microbes in subjects with rCDI.

**Figure 4.**
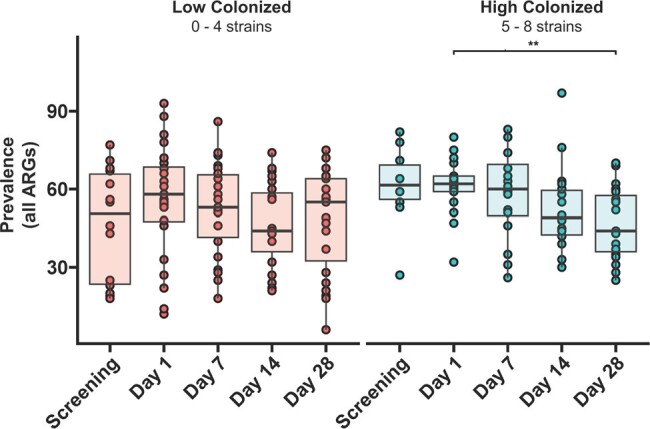
Total ARG prevalence is reduced in subjects with high VE303 colonization (5 to 8 strains colonized) by Day 28. The box-and-whisker plots depict the median, interquartile range (IQR, at the top and bottom of the boxes), and reasonable extreme values at 1.5 X IQR in the dataset (where the vertical lines end). Star and bracket annotations indicate all pairwise comparisons with ARG prevalence at Day 1 that were significantly different; ** p < 0.005, LME model.

**Disclosures:**

**Rajita Menon, PhD**, Vedanta Biosciences: Patent inventor|Vedanta Biosciences: Employment|Vedanta Biosciences: Stocks/Bonds (Private Company) **Emily Crossette, PhD**, Vedanta Biosciences: Employment|Vedanta Biosciences: Stocks/Bonds (Private Company) **Vanni Bucci, PhD**, Vedanta Biosciences: Advisor/Consultant **Marlena Keisler, n/a**, Vedanta Biosciences: Employer **Gregory Medlock, PhD**, Vedanta Biosciences: Ownership Interest **Bernat Olle, PhD**, Vedanta Biosciences, Inc.: Board Member|Vedanta Biosciences, Inc.: Ownership Interest|Vedanta Biosciences, Inc.: Stocks/Bonds (Private Company) **Jeffrey L. Silber, MD**, Vedanta Biosciences: Employee|Vedanta Biosciences: Ownership Interest **Jason Norman, PhD**, Vedanta Biosciences: Patent inventor|Vedanta Biosciences: Employment|Vedanta Biosciences: Stocks/Bonds (Private Company)

